# Impact of Diagnostic Confidence, Perceived Difficulty, and Clinical Experience in Facial Melanoma Detection: Results from a European Multicentric Teledermoscopic Study

**DOI:** 10.3390/cancers17203388

**Published:** 2025-10-21

**Authors:** Alessandra Cartocci, Alessio Luschi, Sofia Lo Conte, Elisa Cinotti, Francesca Farnetani, Aimilios Lallas, John Paoli, Caterina Longo, Elvira Moscarella, Danica Tiodorovic, Ignazio Stanganelli, Mariano Suppa, Emi Dika, Iris Zalaudek, Maria Antonietta Pizzichetta, Jean Luc Perrot, Imma Savarese, Magdalena Żychowska, Giovanni Rubegni, Mario Fruschelli, Ernesto Iadanza, Gabriele Cevenini, Linda Tognetti

**Affiliations:** 1 Dermatology Unit, Department of Medicine, Surgery and Neuroscience, University of Siena, 53100 Siena, Italy; alessandra.cartocci@dbm.unisi.it (A.C.); elisa.cinotti@unisi.it (E.C.); 2 Department of Medical Biotechnologies, University of Siena, 53100 Siena, Italy; alessio.luschi@unisi.it (A.L.); ernesto.iadanza@unisi.it (E.I.); gabriele.cevenini@unisi.it (G.C.); 3 Unit of Diagnostic and Therapeutic Neuroradiology, Department of Neurology and Human Movement Sciences, Azienda Ospedaliero Universitaria, 53100 Siena, Italy; sofia.loconte@student.unisi.it; 4 Department of Dermatology, University of Modena and Reggio Emilia, 41121 Modena, Italylongo.caterina@gmail.com (C.L.); 5 First Department of Dermatology, Aristotle University, 54124 Thessaloniki, Greece; emlallas@gmail.com; 6 Department of Dermatology and Venereology, Institute of Clinical Sciences, Sahlgrenska Academy, University of Gothenburg, Sahlgrenska University Hospital, 41345 Gothenburg, Sweden; john.paoli@vgregion.se; 7 Department of Dermatology and Venereology, Region Vastra Gotaland, Sahlgrenska University Hospital, 41345 Gothenburg, Sweden; 8 Dermatology Unit, University of Campania Luigi Vanvitelli, 80138 Naples, Italy; elvira.moscarella@gmail.com; 9 Dermatology Clinic, Medical Faculty, University of Nis, 18000 Nis, Serbia; danica.dr@gmail.com; 10 Skin Cancer Unit, Scientific Institute of Romagna for the Study of Cancer (IRCSS), 47014 Meldola, Italy; ignazio.stanganelli@irst.emr.it; 11 Department of Dermatology, University of Parma, 43121 Parma, Italy; 12 Department of Dermatology, Hôpital Erasme, Université Libre de Bruxelles, 1050 Brussels, Belgium; mariano.suppa@erasme.ulb.ac.be; 13 Groupe d’Imagerie Cutanée Noninvasive, Société Française de Dermatologie, 75009 Paris, France; 14 Department of Dermatology, Institut Jules Bordet, 1070 Brussels, Belgium; 15 Oncologic Dermatology Unit, IRCCS Azienda Ospedaliero Universitaria di Bologna, 40126 Bologna, Italy; emi.dika3@unibo.it; 16 Oncologic Dermatology, Department of Medical and Surgical Sciences, University of Bologna, 40138 Bologna, Italy; 17 Department of Dermatology and Venereology, University of Trieste, 34141 Trieste, Italy; iris.zalaudek@gmail.com (I.Z.); pizzichetta@cro.it (M.A.P.); 18 Department of Medical Oncology, Centro di Riferimento Oncologico di Aviano (CRO), IRCSS, 33081 Aviano, Italy; 19 Dermatology Unit, University Hospital of St-Etienne, 42270 Saint Etienne, France; j.luc.perrot@chu-st-etienne.fr; 20 Soc Dermatologia Pistoia-Prato, USL Toscana Centro, 51100 Pistoia, Italy; imma.savarese@uslcentro.toscana.it; 21 Department of Dermatology, Faculty of Medicine, University of Rzeszow, 35-310 Rzeszow, Poland; magda.zychowska@gmail.com; 22 Ophthalmology Unit, Department of Medicine, Surgery and Neurosciences, University of Siena, 53100 Siena, Italy; giovanni.rubegni@unisi.it (G.R.); mario.fruschelli@unisi.it (M.F.)

**Keywords:** dermoscopy, atypical pigmented facial lesions, lentigo maligna, pigmented actinic keratosis, solar lentigo, diagnostic accuracy, diagnostic confidence

## Abstract

Background: Diagnosing facial melanoma, specifically lentigo maligna (LM) and lentigo maligna melanoma (LMM), is a daily clinical challenge, particularly for small or traumatized lesions. LM and LMM are part of the broader group of atypical pigmented facial lesions (aPFLs), which also includes benign look-alikes such as solar lentigo (SL), atypical nevi (AN), seborrheic keratosis (SK), and seborrheic-lichenoid keratosis (SLK), as well as pigmented actinic keratosis (PAK), a potentially premalignant keratinocytic lesion. Standard dermoscopy with handheld devices is the most widely used diagnostic tool in dermatology, but its accuracy heavily depends on the clinician’s experience and the perceived difficulty of the case. As a result, many benign aPFLs are excised for histological analysis, often leading to aesthetic concerns. Reflectance confocal microscopy (RCM) can reduce the need for biopsies, but it is limited to specialized centers and requires skilled operators. Aims: This study aimed to assess the impact of personal skill, diagnostic confidence, and perceived difficulty on the diagnostic accuracy and management in the differential dermoscopic diagnosis of aPFLs. Methods: A total of 1197 aPFLs dermoscopic images were examined on a teledermoscopic web platform by 155 dermatologists and residents with 4 skill levels (<1, 1–4, 5–8, >8 years). They were asked to give a diagnosis, to estimate their confidence and rate the case, and choose a management strategy: “follow-up”, “RCM” or “biopsy”. Diagnostic accuracy was examined according to the personal skill level, confidence level, and rating in three settings: (I) all seven diagnoses, (II) LM vs. PAK vs. fully benign aPFLs, (III) malignant vs benign aPFLs. The same analyses were performed for management decisions. Results: The diagnostic confidence has a certain impact on the diagnostic accuracy, both in terms of multi-class diagnosis of six aPFLs in diagnostic (setting 1) and in benign vs malignant (setting 3) or benign vs. malignant/premalignant discrimination (setting 2). The perceived difficulty influences the management of benign lesions, with easy ratings predominantly matching with “follow-up” decision in benign cases, but not that of malignant lesions assigned to “biopsy”. The experience level had an impact on the perception of the number of real easy cases and had no to minimal impact on the average diagnostic accuracy of aPFLs. It, however, has an impact on the management strategy and specifically in terms of error reduction, namely the lowest rates of missed malignant cases after 8 years of experience and the lowest rates of inappropriate biopsies of benign lesions after 1 year of experience. Conclusions: The noninvasive diagnosis and management of aPFLs rest on a daily challenge. Highlighting which specific subgroups of lesions need attention and second-level examination (RCM) or biopsy can help detect early malignant cases, and, in parallel, reduce the rate of unnecessary removal of benign lesions.

## Simple Summary

The dermoscopic differential diagnosis of pigmented facial lesions poses a daily challenge, and lentigo maligna can be simulated by a series of beginning entities, especially small ones, such as pigmented actinic keratosis or solar lentigo. While it is known that personal dermoscopic skill largely relies on clinical experience, the level of diagnostic confidence and perceived difficulty of those cases have never been investigated. Here, we highlighted that diagnostic confidence has a certain impact on the diagnostic accuracy of benign and malignant difficult pigmented facial lesions, while perceived difficulty seems to influence management more. A higher personal experience in dermoscopy has a greater impact on management strategies and the recognition of easy cases than on the average diagnostic accuracy of aPFLs.

## 1. Introduction

### 1.1. Facial Melanoma

Malignant melanoma (MM) is a major public health issue worldwide, with incidence doubling in the past decade. In fair-skinned populations, annual rates are about 25 to 30 per 100,000 in Europe and the United States, and up to 60 per 100,000 in Australia [1]. Globally, incidence is projected to rise by more than 50% from 2020 to 2040 (325,000 to 510,000 new cases each year), with a 68% increase in mortality [2]. Facial subtypes such as lentigo maligna (LM) and lentigo maligna melanoma (LMM) are becoming more frequent due to population ageing and cumulative ultraviolet exposure [3]. Early detection is challenging because atypical pigmented facial lesions (aPFLs) often mimic melanoma and include benign and premalignant entities such as solar lentigo and pigmented actinic keratosis [4].

### 1.2. Melanoma Dermoscopic Diagnosis

Dermoscopy improves diagnostic accuracy compared to direct and unaided diagnosis, especially for experienced examiners. It increases diagnostic odds for pigmented lesions by nearly 50% relative to naked eye assessment, with expert users reaching sensitivities near ∼90% and specificities around 59%, compared with roughly 60% accuracy without dermoscopy [5,6]. Novices benefit less, highlighting the need for structured training [7]. Dermoscopy can push melanoma detection accuracy to about 90%, but gains are greatest for experts, while novices commonly do not exceed naked eye accuracy without formal training to build skill and confidence [8]. The effect of experience has been assessed in novice clinicians. First-year dermatology residents completing brief online modules reported significant improvements in confidence and performance [9]. Vestergaard et al. [10] showed that teledermoscopy achieved sensitivity similar to face-to-face evaluation but lower specificity, with higher diagnostic confidence in person. Accuracy improved with photo quality and years of experience, and complete confidence in teledermoscopic decisions aligned with correct management in all melanoma cases. In remote triage, Rogers et al. [11] found that adding dermoscopic to clinical images improved malignancy recognition by 23%, increased correct diagnoses from 45% to 53%, and raised confidence by 7.6%. Confidence correlated positively with correctness, although image quality and variable training limited consistency. Earlier work noted that confidence often overestimates accuracy with substantial interobserver variability, but dermoscopy helps align confidence with true performance for pigmented lesions. Differences in dermoscopic patterns between simulators and malignancies also influence both accuracy and certainty [12,13].

### 1.3. Differential Dermoscopic Diagnosis of Facial Melanoma

For facial pigmented lesions, traditional pattern analysis yields modest sensitivity (∼56.7%), particularly in early or small tumors. In contrast, a recently proposed inverse approach that focuses on the absence of features typical of pigmented actinic keratosis or solar lentigo significantly improves early LM detection [14]. After targeted training, reader sensitivity rose to 83.6% and overall correct classification increased from 65.5% to 78.5%; more than half of readers outperformed a convolutional neural network trained on the same data [14]. Educational strategies, including dermoscopy masterclasses and AI-assisted learning, further enhance performance, with stepwise training improving sensitivity and accuracy [7,9,14]. Although dedicated face simulators have not been assessed, image-based training environments function as effective diagnostic simulators for complex facial lesions. Overall, dermoscopy shows modest accuracy for distinguishing malignant from benign aPFLs, especially in small, early, or traumatized lesions. In elderly patients with diffusely sun-damaged facial skin, detection is difficult in routine practice amid rising patient volumes. Malignant facial melanoma (LM and LMM) is frequently simulated by common benign lesions such as solar lentigo (SL), seborrheic keratosis (SK), and seborrheic-lichenoid keratosis (SLK). Two premalignant keratinocytic entities, pigmented actinic keratosis (PAK) and atypical nevi (AN), also contribute to diagnostic confusion [3,4]. Reflectance confocal microscopy (RCM) is a valuable adjunct for these difficult cases. RCM shows higher sensitivity (∼80%) and more consistent interobserver agreement, with slightly lower specificity (∼81%) than dermoscopy (∼61% sensitivity, 92% specificity) [15,16]. RCM is operator-dependent, available in a few centers, time-consuming, and limited by issues such as hyperkeratosis or bleeding.

### 1.4. Objectives

To the authors’ knowledge, no prior study has quantified how dermatologists’ subjective confidence and objective experience influence diagnostic and management accuracy for facial LM or LMM lesions. Previous studies by our group highlighted that the level of experience in dermoscopy can play a role in the teledermoscopic diagnosis of cases of aPFL [3,4,17]. Our first objective was to assess how the perceived difficulty of the case, the confidence in the diagnosis, and the experience in dermoscopy, in a teledermatoscopic setting, affect the diagnosis and management of malignant lesions. Second, we aimed to evaluate the specific role of these three variables in determining the final diagnostic accuracy, either in three settings: (i) a benign/malignant discrimination of aPFLs; (ii) a multi-diagnosis of the six above-mentioned aPFLs (LM/LMM vs. PAK vs. AN vs. SL vs. SK vs. SLK); (iii) a differential diagnosis of benign vs. premalignant vs. malignant aPFLs.

## 2. Materials and Methods

### 2.1. Study Design

The study was carried out as part of the iDScore (Integrated Dermoscopy Scoring system) facial project. The project has been carried out in accordance with the Helsinki Declaration and was approved by the local Ethics Committee (Study Protocol No. 16801). All personal data were de-identified before use. The project was performed by the iDScore team of dermatologists and scientists of Siena University Hospital in collaboration with the Teledermoscopy Working Group of the EADV Teledermatology Task Force.

### 2.2. Study Dataset

The present study is based on the iDScore-facial database, a curated collection comprising 1197 dermoscopic images of histopathologically confirmed pigmented facial lesions, including 499 LM/LMM, 231 PAK, 266 SL, 48 SK, 28 SLK, and 125 AN, as described in Tognetti et al. [18]. All lesions were located at “orbital area”, “forehead”, “nose”, “cheek”, “chin” or “mouth”.

### 2.3. Teledermoscopic Test

All cases were then independently evaluated on the iDScore web platform by a total of 157 dermatologists, generating a cumulative total of 2445 evaluations.

All participants were required to declare their experience level in dermoscopy in terms of years of continuous use of a dermatoscope in their clinical practice: they were thus stratified according to four skill levels, namely level I = <1 year, level II = 1–4 years, level III = 5–8 years, and level IV = >8 years.

Each evaluation, instead, consisted of a direct diagnosis of the lesion (the options were: LM/LMM, PAK, SL, SK, SLK, and AN) with a management strategy (the options were: close dermoscopic follow-up, additional noninvasive diagnostic examination such as reflectance confocal microscopy, or performing a skin biopsy), a dermoscopic pattern analysis, and an assessment of participant’s diagnostic confidence (i.e., level of confidence) and the perceived difficulty of the case (i.e., case rating).

The confidence level was assessed using a 5-point Likert scale: “not confident” (1), “mildly underconfident” (2), “uncertain” (3), “mildly confident” (4), and “very confident” (5). Then, participants were guided to assess the case rating, still using a 5-point Likert scale, ranging from “very easy” (1), “easy” (2), “moderate” (3), “difficult” (4), and “very difficult” (5).

### 2.4. Statistical Analysis

Descriptive statistics were computed to summarize the characteristics of the dataset. For categorical variables, absolute frequencies and percentages were reported. For quantitative variables, mean and standard deviation (SD) were reported.

To assess the association between dermatologist experience level, diagnostic confidence, and perceived difficulty of the cases, Chi-squared tests were performed.

Diagnostic performance was evaluated by estimating the accuracy and the sensitivity of LM/LMM. The analyses were also repeated stratifying by the different diagnostic settings described below and by participants’ experience, confidence level, and case rating. Their 95% confidence intervals were estimated. Krippendorf’s alpha and 95% confidence intervals were estimated to evaluate the inter-rater reliability.

To facilitate robust statistical analyses and ensure adequate sample sizes within each subgroup, both Likert scales (confidence level and case rating) were recoded into three-level categorical variables. Confidence scores were recoded as follows: scores of 1 or 2 were classified as “not confident”, a score of 3 as “uncertain”, and scores of 4 or 5 as “confident”. A similar recoding procedure was applied to the perceived difficulty variable: scores of 1 or 2 were labelled “difficult”, a score of 3 as “moderate”, and scores of 4 or 5 as “easy”.

The diagnostic performance of dermatologists was assessed in three different classification settings designed to reflect different clinical decision-making scenarios. In Setting 1, a full six-category classification was maintained (LM/LMM, AN, PAK, SL, SK, SLK). This setting aimed to retain maximum granularity and specificity. In Setting 2, diagnoses were grouped into three clinically relevant categories: malignant lesions (LM + LMM), premalignant lesions (PAK), and benign lesions (SL, SK, SLK, AN). Setting 3 further simplified the classification task into a binary model, comparing malignant lesions (LM + LMM) against all other lesions grouped as benign or premalignant (AN, PAK, SL, SK, SLK), thereby simulating a real-world scenario.

A *p*-value of less than 0.05 was considered statistically significant. All statistical analyses were conducted using R (version 4.3.1).

## 3. Results

The study included 157 participants, of whom 53.5% were female (n = 85) and 46.5% male (n = 72). The average age was 36.5 years (SD = 10.6, range 24–67). Most participants completed their education in Italy (78%), while 22% in other European countries. In terms of profession, 67% were dermatologists (n = 105) and 33% dermatology residents (n = 52). Regarding experience in dermoscopy, 22.1% had <1 year, 28.6% between 1 and 4 years, 14.3% between 5 and 8 years, and 36.4% >8 years. A total of 63.6% of the participants performed tests on laptops, 29.9% on smartphones, and the remaining on tablets.

### 3.1. Correlation Between Confidence Level, Perceived Difficulty, and Experience in Dermoscopy

**Figure 1:**
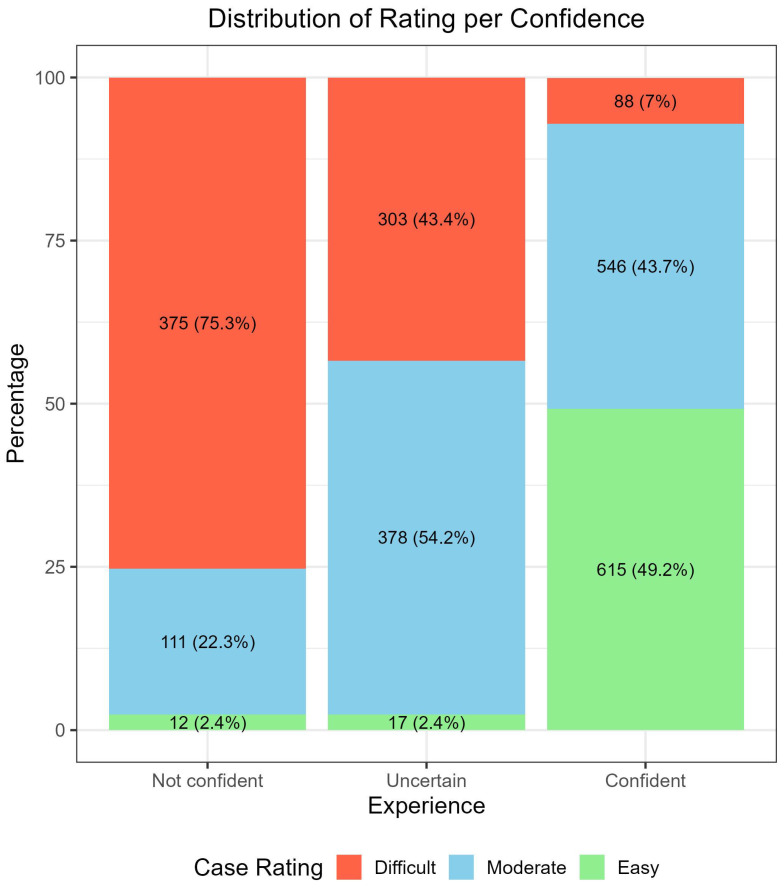
Distribution of the case rating (Easy, Moderate, and Difficult) across evaluators’ confidence in the assessment.

Figure 1 shows the distribution of difficulty ratings (“difficult,” “moderate,” “easy”) across confidence levels (“not confident,” “uncertain,” “confident”). When participants were not confident, most cases (75.3%) were rated as difficult. In the “uncertain” group, 54.2% of cases were considered moderately difficult and 43.4% were difficult. When participants were confident, nearly half of the cases were rated as easy. These distributions were significantly associated (*p* < 0.001).

**Figure 2:**
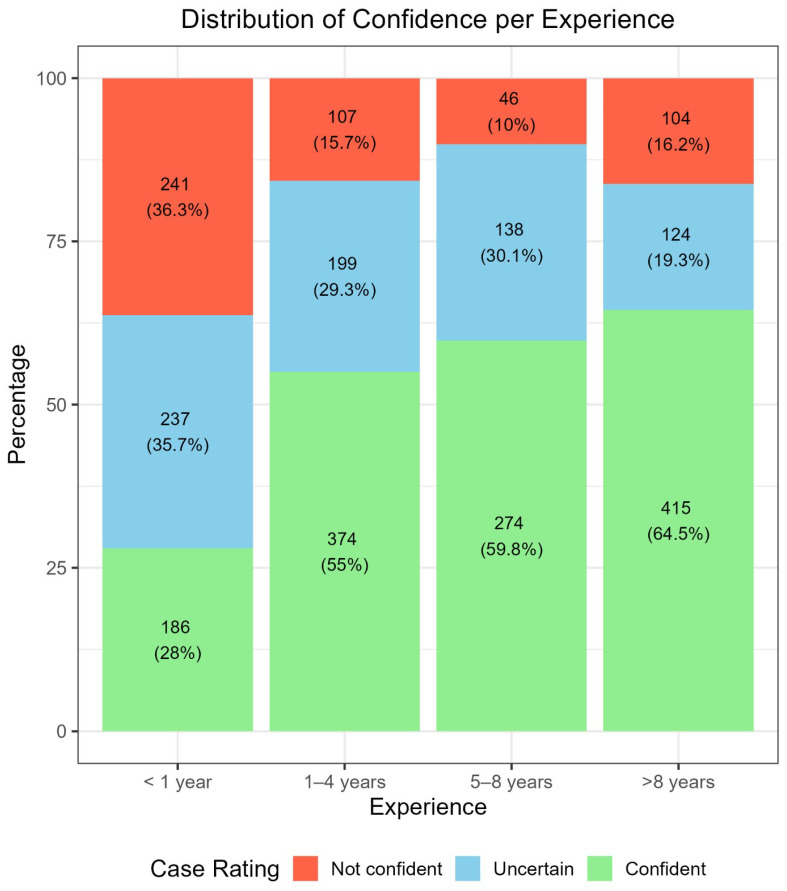
Distribution of the confidence in the assessment (Not confident, Uncertain, Confident) across evaluators’ experience level.

Figure 2 displays confidence ratings across experience levels. Confidence increased steadily with years of dermoscopy practice, ranging from 28% in group <1 year to 64.5% in group >8 years. Conversely, “not confident” ratings decreased from 36.3% in the first group to 10% in the 5–8 years group, with a slight rebound in the group of >8 years (16.2%). These associations were statistically significant (*p* < 0.001).

**Figure 3:**
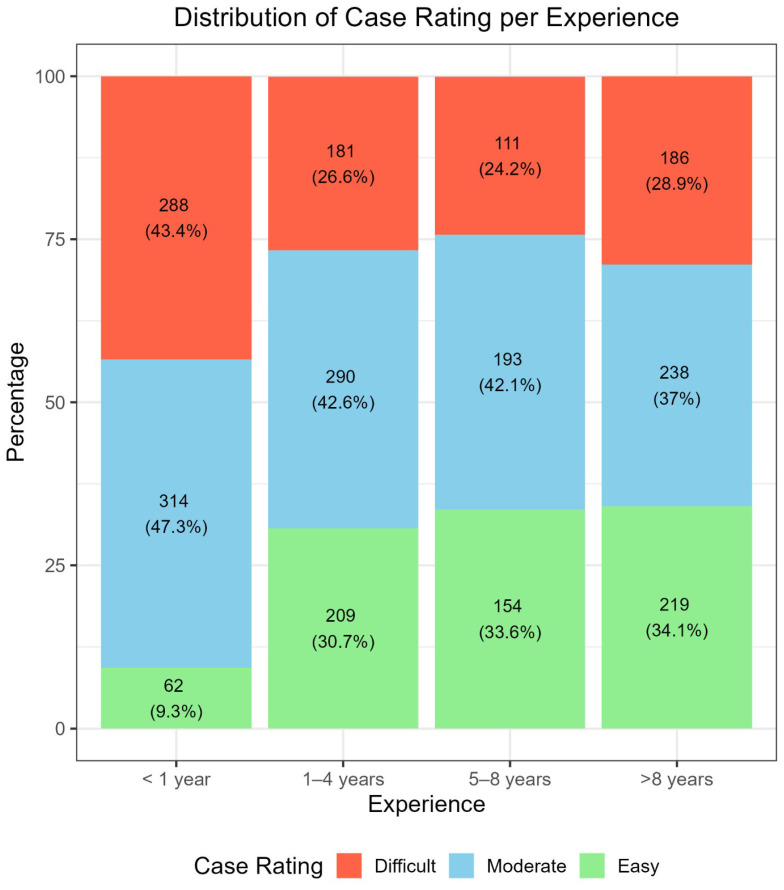
Distribution of the case rating (Easy, Moderate, and Difficult) across evaluators’ experience level.

Figure 3 illustrates difficulty ratings across experience levels. The group with higher experience showed similar distributions of “difficult,” “moderate,” and “easy” cases, reflecting the case mix and teledermoscopic setting. In contrast, Group I (<1 year of experience) differed markedly, showing nearly twice as many “difficult” ratings, more “moderately difficult” ratings, and about one quarter fewer “easy” ratings. The association between difficulty and experience was significant (*p* < 0.001).

### 3.2. Diagnostic Accuracy over 3 Testing Sets: Comparison of Relative Impact of Experience, Confidence, and Difficulty Rating

Diagnostic accuracy on aPFLs varied substantially across the three evaluated clinical settings. Setting 1, which included a broader mix of lesions (LM/LMM, AN, PAK, SL, SK, SLK), demonstrated the lowest overall accuracy at 48.7%. Accuracy improved in Setting 2 (60.0%), and was highest in Setting 3 (71.2%), where the diagnostic comparison was limited to LM/LMM and benign lesions (Table 1). Krippendorf’s alpha values for the overall inter-rater agreement are 0.36, 0.36, and 0.37 for the three settings, respectively.

Stratifying by clinician experience, those with less than 1 year of practice exhibited slightly lower accuracy in Setting 1 (47.7%) compared to more experienced clinicians. However, across all experience levels, accuracy increased consistently from Setting 1 to Setting 3, indicating that the restriction of differential diagnoses had a universally positive effect. For example, clinicians with 1–4 years of experience improved from 49.9% in Setting 1 to 71.5% in Setting 3. Those with more than 8 years of experience demonstrated a similar trend, with accuracy rising from 49.1% to 71.1% (Table 1). Confidence in diagnostic decisions was strongly associated with accuracy. Participants who were mildly or very confident achieved 52.8%, 66.4%, and 78.1% accuracy in Settings 1, 2, and 3, respectively. Conversely, those who reported being not confident or mildly underconfident had lower accuracy (40.2% in Setting 1 and 61.4% in Setting 3). An uncertain confidence level yielded intermediate performance, reaching 65.9% in Setting 3 (Table 1). Case difficulty, as rated by the participants, also correlated with performance. Accuracy for cases rated as “easy” was markedly higher than for “moderate” or “difficult” ones. In Setting 3, easy cases were associated with 82.3% accuracy, compared to 70.5% and 62.9% for moderate and difficult cases, respectively. This pattern held across all settings (Table 1).

### 3.3. Specific Impact of Confidence Level on the Diagnostic Performance, Based on the Personal Experience

Table 2 reports the accuracy and sensitivity values obtained on the three testing subsets, grouped according to personal experience and confidence level. Again, setting 3 of testing yielded the higher accuracy values, on average. Of note, the cases that received a high-confidence assessment in the four age-groups reached very similar diagnostic accuracy values (range 75.9–79.9%). On average, we can observe a proportional trend between accuracy values and experience level towards all groups, regardless of experience level. Participants proposed the correct diagnosis, especially that of malignant cases, in those cases where they appear more confident, even if they have very different experience levels.

### 3.4. Specific Impact of Perceived Difficulty on the Diagnostic Performance, Based on the Personal Experience

Table 3 reports the accuracy and sensitivity values obtained on the three testing subsets, grouped according to personal experience and perceived difficulty. On average, the case rating seems to impact more than the clinical personal experience on the final diagnostic accuracy, e.g., 79 and 82.6% of accuracy were obtained on lesions judged “easy” by skill level I and III participants, respectively, in setting 3. Of note, higher sensitivity values appear to correlate with “easy” rating only in lesions examined by II and IV (i.e., 65.2 and 64.8%). Of note, the highest SE values for lesions evaluated by groups I and III were observed in the cases judged “difficult” (59.5 and 58%).

### 3.5. Impact of Confidence Level Plus Perceived Case Difficulty on the Diagnostic Performance

Table A1 illustrates the results of the cross-analysis carried out to highlight the variation of diagnostic accuracy values according to skill level, confidence degree, and case difficulty, on the same aPFLs lesions. Taking into account the case distribution, we can appreciate that the case rating and the assessment of confidence level are proportionally matched in many cases. On one side, in less experienced participants (<1 year of experience), the most numerous lesion group is the one matching “not confident” and “difficult”, i.e., 186 cases. On the other side, the groups matching “confident” and “easy” are the most numerous in the other three participant groups, namely: 200 in skill level II (1–4 years), 153 in skill level III (i.e., 5–8 years), and 208 in skill level IV (i.e., >8 years). Conversely, the diagnostic accuracy (in settings 1 to 3) does not seem to correlate with the confidence degree, but is somehow in line with the case difficulty rating. Many lesions considered “easy” were obtained 100%. Accuracy in diagnostic settings 2 and 3, although participants from skill levels I, III, and IV admitted being “not confident” (Table A1).

### 3.6. Impact of Dermoscopic Experience, Confidence Level, and Difficulty Rating on aPFLs Management Strategies

All management actions proposed by participants over the whole case study are reported in Table 4 and independently grouped by experience level, confidence, or difficulty rating. This descriptive analysis illustrates that among aPFLs assigned to close dermoscopic FU, about 70% were judged “easy” and with a high confidence level (57.2% of cases); among aPFLs assigned to RCM, 38% were difficult and with an “uncertain” confidence degree (33.2%); among aPFLs assigned to skin biopsy, 44.4% were judged difficult and with an “uncertain” degree of confidence (42.4%). In general, the easiest cases were assigned to FU, while the more difficult cases to biopsy, which is always more frequently indicated than RCM, even in easy and uncertain cases.

### 3.7. Impact of Dermoscopic Experience, Confidence Level, and Difficulty Rating on Management Strategies of Malignant and Benign aPFLs

Management approaches varied significantly between benign lesions and LM/LMM, with clear patterns observed across levels of experience, diagnostic confidence, and perceived case difficulty.

Analyzing the findings according to personal skill level, it appeared that: in benign cases, clinicians of all experience levels predominantly chose close dermoscopic follow-up, ranging from 45.8% to 53.8%; in malignant cases, the preferred strategy was consistently biopsy in all skill groups (range 38.5–63.6%). Conversely, rates of RCM suggestion are very similar in both benign and malignant cases, regardless of the skill level.

Considering the expressed confidence level, it appeared that: in benign cases, confident clinicians preferred follow-up (57.6%), whereas uncertain and not confident clinicians used RCM more (35.7% and 42.4%, respectively) and followed up less (30.2% and 36.0%); in malignant cases, biopsy was the most frequent choice across all confidence levels (50.5–53.8%); notably, RCM was mostly used on benign cases when clinicians were not confident (42.4% of cases).

Based on difficulty case rating, it appeared that: in benign lesions, follow-up was dominant in easy cases (80.0%), but decreased as difficulty increased (22.6% in difficult cases), while RCM again in difficult benign cases (42.3%); in malignant cases, biopsy rates were somehow similar in easy (53.8%) and difficult (55.4%) cases, while RCM for malignant lesions was choose in only 33% of cases (Table 5).

**Figure 4:**
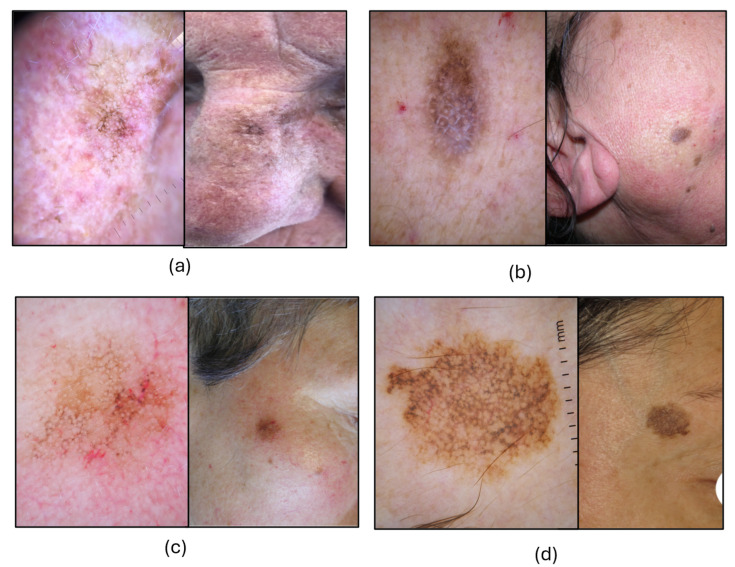
Dermoscopic and clinical images of four atypical pigmented facial lesions (aPFLs) diagnosed blindly on a teledermoscopic setting with various confidence and difficulty perception levels. (**a**) Lesion on the nose evaluated by a dermatology resident with less than four years of experience in dermoscopy, rated “easy”, diagnosed as PAK, being “very confident”: histologic diagnosis was PAK. (**b**) Lesion on the cheek evaluated by a dermatology resident with less than one year of experience, rated “moderately difficult”, and diagnosed SLK, being “uncertain”: histologic diagnosis was PAK. (**c**) Lesion on the orbital area evaluated by a dermatologist with five to eight years of experience, rated “difficult” and diagnosed as SK, being “mildly confident”: histology was LM. (**d**) Lesion on the orbital area evaluated by a dermatologist with more than eight years of experience, rated “very difficult” and diagnosed as SLK, being “not confident”. Histology was LM.

Figure 4 illustrates some indicative aPFL cases examined by differently experienced clinicians on the web platform, in which three different confidence levels correspond, each, to easy or difficult ratings and to a correct or wrong diagnosis, respectively, demonstrating one more the challenging nature of this teledermoscopic diagnosis.

**Figure 5:**
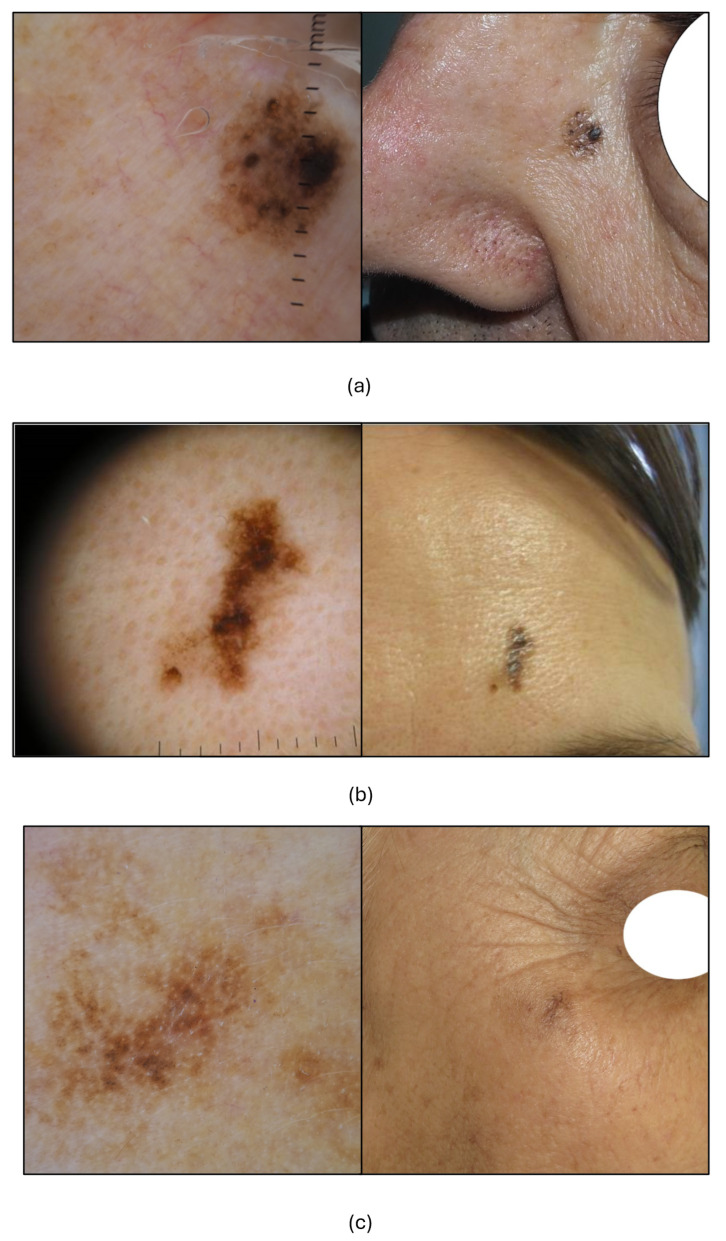
Inappropriate management of three aPFLs examined blindly on a teledermoscopic setting influenced by the confidence and difficulty perception levels. (**a**) Lesion on the nose evaluated by a dermatology resident with less than one year of experience in dermoscopy, rated “very difficult” and diagnosed as SK being “not confident”, assigned to close follow-up: histologic diagnosis was LM. (**b**) Lesion on the front evaluated by dermatology with more than eight years of experience, rated “moderately difficult”, and diagnosed AN as “uncertain”, assigned to RCM: histologic diagnosis was LMM. (**c**) The lesion on the orbital area was evaluated by a dermatologist with more than eight years of experience, rated “difficult” and diagnosed as LM, being “mildly underconfident” and assigned to biopsy. Histology was SL.

Figure 5 illustrates three cases of inappropriate management and low to poor confidence and rating, proposed by clinicians of different experience in dermoscopy.

## 4. Discussion

Although dermoscopy has been extensively studied across settings, and remains indispensable for facial melanoma screening and early detection, the roles of diagnostic confidence and perceived difficulty are less clearly defined [19,20,21]. Evidence indicates that while confidence generally increases with use and experience, it does not always equal accuracy. Structured training, high-quality imaging, and careful recognition of simulators are essential to ensure that high confidence aligns with correct decisions, especially in teledermatology and triage [5,9,10,16]. RCM provides complementary value by mitigating observer variability and improving reliability for difficult facial lesions [22,23,24]. This study is the first to demonstrate the effects of diagnostic confidence, difficulty rating, and personal experience on teledermoscopic diagnosis and management of anonymized aPFLs, both benign and malignant.

Overall, confidence and difficulty ratings tended to be positively correlated. Low confidence often coincided with higher difficulty (about two thirds of cases), while easy ratings paired with high confidence in about one third (Figure 1). However, when stratified by experience (Table A1), the combination of high confidence and easy rating did not always yield the best accuracy across settings and skill levels. Likewise, not confident and difficult did not always correspond to the worst accuracy. Thus, confidence affects accuracy, but difficulty rating appears to exert a stronger influence in teledermoscopy [3,4,25].

Confidence increased with years of dermoscopy practice in the six-category task (Figure 2), with a small rise in not confident cases after more than eight years. This mirrors clinical behavior where experience can lead to a cautious approach influenced by memorable difficult cases, bringing us to speculate a kind of “experience bias” [3,5,11,20,26]. Greater confidence is associated with higher diagnostic accuracy, especially for malignant versus benign classification (Table 1). When grouped by skill (Table 2), this trend did not fully align across all settings, suggesting that confidence exerts more influence than experience in this context. Confidence also shaped management: when confident, participants chose follow-up in 57.2% of cases. When not confident, biopsy and RCM had similar proportions (39.8% and 36.1%) (Table 4). Specifically, confident participants most often chose appropriate follow-up for benign cases and biopsy for malignant ones (57.6% and 53.8%) (Table 5), with RCM used across both groups.

Perceived difficulty varied with experience. Easy judgments increased with practice (Figure 2), while moderate judgments were similar between less experienced (levels I and II) and more experienced (levels III and IV) clinicians. After the first year, difficult judgments decreased and then remained stable (range 26.6% to 28.9%) (Figure 3). Dermoscopy therefore helps recognize easy benign facial lesions (most SL, SK, SLK and some PAK or AN), while about one quarter of aPFLs remain challenging, especially in teledermoscopy. Accuracy rose with easier ratings in all three settings, with gaps near 10% in Setting 1, 15% in Setting 2, and 20% in Setting 3 (Table 1). After stratifying by experience, differences were minimal (Table 3). Interestingly, experts (level IV) performed relatively worse on lesions they judged difficult across all settings, suggesting some influence of self-confidence in teledermoscopy compared with in-person assessment [10,25,27,28,29]. Perceived difficulty also guided management. Easy cases were usually followed up, and difficult cases were more often biopsied, largely independent of experience. Nearly 70% of cases rated easy were sent to follow-up (Table 4), and among benign cases specifically, 80% of easy lesions were followed up (Table 5). For difficult benign cases, RCM and biopsy were suggested at similar rates (42.3% and 35%). For malignant cases, biopsy rates were similar for easy, moderate, and difficult lesions (about 53% to 55%). Considering errors, experts (level IV) had the lowest rate of follow-up in malignant cases (19% versus 22%, 23%, and 31% in other levels), while level II showed the lowest rate of biopsy for benign cases (18.9% versus 23% and 31%). This indicates that clinicians may perform better in management than in diagnosis for these difficult lesions in teledermoscopy [25,27,28,29].

### Limitations

This study presents several limitations that may impact the generalizability and applicability of its findings, usually related to web-based teledermoscopic studies. First, the assessments were conducted outside of a real-world clinical setting, potentially limiting the integration of contextual cues such as patient history, lesion palpability, or visual examination under natural lighting. Second, the use of web-based assessments may have introduced participant fatigue, particularly during lengthy evaluation sessions, potentially affecting concentration and diagnostic performance. Third, while the use of a large and curated dermoscopic dataset of images ensured diagnostic standardization, the reliance on simulated cases rather than live patient interactions may not fully reflect real-world clinical variability. Moreover, image quality varied across cases, and although this mirrors real teledermoscopic conditions, it may have influenced diagnostic confidence and accuracy. Finally, the unequal distribution of participants across skill levels may have affected the statistical power of subgroup comparisons and limited the interpretation of experience-related trends.

## 5. Conclusions

In the teledermoscopic evaluation of 1197 aPFLs, diagnostic confidence influenced accuracy in both the six-category task and the benign versus malignant or benign versus malignant versus premalignant scenarios. Difficulty ratings shaped management of benign lesions, with easy ratings aligning with follow-up, whereas biopsy for malignant cases was largely independent of difficulty. Experience affected the proportion of truly easy cases identified. Dermoscopic experience had minimal effect on average diagnostic accuracy for aPFLs, with only about a 2% difference between novices (less than one year) and experts (more than eight years) in Settings 1 and 2, and identical values in Setting 3. This suggests that, beyond the intrinsic difficulty of facial lesions, younger dermatologists may be more comfortable with teledermoscopy and benefit from pattern analysis tools on the web platform [3,4,19,25,28]. Experience became more evident in management choices, especially after eight years, with fewer errors, such as assigning malignant cases to follow up rather than biopsy or RCM.

Targeted training on aPFLs should be included in residency curricula to address the growing burden of these conditions in ageing, photodamaged populations. Training should cover LM or LMM [30,31,32,33], PAK [34,35], atypical SL [36,37], SLK [37,38], SK [39], and AN [40]. A teledermoscopic web platform is a practical tool to reach residents in centers, mitigate limited clinical exposure, provide curated teaching cases, and strengthen personal confidence. Future studies should incorporate real-time clinical assessments, explore the impact of image quality standardization, and aim for more balanced participant recruitment across experience strata. Longitudinal designs and the inclusion of patient-level outcomes may further enhance the ecological validity and clinical relevance of such investigations.

## Figures and Tables

**Table 1:** Diagnostic accuracy results obtained from teledermoscopic evaluations of 1197 atypical pigmented facial lesions (aPFLs) in three different settings, stratified across levels of experience in dermoscopy.

		N	Setting 1 ^1^ (%)	Setting 2 ^2^ (%)	Setting 3 ^3^ (%)
Overall		2445	48.7 (46.7, 50.7)	60.0 (58.0, 61.9)	71.2 (69.4, 73.0)
Experience	<1 year	664	47.7 (43.9, 51.6)	59.5 (55.6, 63.2)	71.4 (67.8, 74.8)
	1–4 years	680	49.9 (46.0, 53.7)	60.6 (56.8, 64.3)	71.5 (67.9, 74.8)
	5–8 years	458	47.8 (43.2, 52.5)	57.6 (53.0, 62.2)	71.0 (66.6, 75.1)
	>8 years	643	49.1 (45.2, 53.1)	61.4 (57.6, 65.2)	71.1 (67.4, 74.6)
Confidence	Not confident	498	40.2 (35.8, 44.6)	48.8 (44.3, 53.3)	61.4 (57.0, 65.7)
	Uncertain	698	47.4 (43.7, 51.2)	56.3 (52.5, 60.0)	65.9 (62.2, 69.4)
	Confident	1249	52.8 (50.0, 55.6)	66.4 (63.8, 69.1)	78.1 (75.7, 80.4)
Rating	Difficult	766	43.9 (40.3, 47.5)	52.1 (48.5, 55.7)	62.9 (59.4, 66.4)
	Moderate	1035	48.5 (45.4, 51.6)	59.9 (56.8, 62.9)	70.5 (67.6, 73.3)
	Easy	644	54.8 (50.9, 58.7)	69.4 (65.7, 72.9)	82.3 (79.1, 85.2)

^1^ LM + LMM vs. AN vs. PAK vs. SL vs. SK vs. SLK diagnosis. ^2^ LM + LMM diagnosis vs. PAK diagnosis vs. BKL + AN diagnosis. ^3^ LM + LMM diagnosis vs. Benign aPFLs diagnosis.

**Table 2:** Diagnostic accuracy results obtained from teledermoscopic evaluations of 1197 atypical pigmented facial lesions (aPFLs) in three different settings, stratified across levels of experience in dermoscopy and confidence of the assessment.

Skill Level	Confidence Level	N	Accuracy Setting 1 ^1^ (%)	Accuracy Setting 2 ^2^ (%)	Accuracy Setting 3 ^3^ (%)	Sensitivity (%)
<1 year	Not confident	241	44.0 (37.6, 50.5)	52.7 (46.2, 59.1)	68.0 (61.8, 73.9)	55.9 (45.2, 66.2)
	Uncertain	237	51.9 (45.3, 58.4)	62.4 (56.0, 68.6)	70.9 (64.6, 76.6)	59.5 (49.7, 68.7)
	Confident	186	47.3 (40.0, 54.7)	64.5 (57.2, 71.4)	76.3 (69.6, 82.3)	51.9 (37.8, 65.6)
1–4 years	Not confident	107	32.7 (24.0, 42.5)	41.1 (31.7, 51.0)	51.4 (41.5, 61.2)	38.3 (24.5, 53.6)
	Uncertain	199	48.2 (41.1, 55.4)	55.8 (48.6, 62.8)	66.3 (59.3, 72.9)	50.6 (39.6, 61.5)
	Confident	374	55.6 (50.4, 60.7)	68.7 (63.8, 73.4)	79.9 (75.5, 83.9)	61.3 (52.6, 69.5)
5–8 years	Not confident	46	45.7 (30.9, 61.0)	50.0 (34.9, 65.1)	58.7 (43.2, 73.0)	51.9 (31.9, 71.3)
	Uncertain	138	46.4 (37.9, 55.1)	53.6 (44.9, 62.1)	65.2 (56.6, 73.1)	52.6 (38.9, 66.0)
	Confident	274	48.9 (42.4, 55.0)	60.9 (54.9, 66.8)	75.9 (70.4, 80.8)	31.2 (21.0, 42.7)
>8 years	Not confident	104	36.5 (27.3, 46.5)	47.1 (37.2, 57.1)	57.7 (47.6, 67.3)	48.8 (33.3, 64.5)
	Uncertain	124	38.7 (30.1, 47.9)	48.4 (39.3, 57.5)	56.5 (47.3, 65.3)	45.2 (32.5, 58.3)
	Confident	415	55.4 (50.5, 60.3)	68.9 (64.2, 73.3)	78.8 (74.5, 82.6)	63.9 (55.9, 71.4)

^1^ LM + LMM vs. AN vs. PAK vs. SL vs. SK vs. SLK diagnosis. ^2^ LM + LMM diagnosis vs. PAK diagnosis vs. BKL + AN diagnosis. ^3^ LM + LMM diagnosis vs. Benign aPFLs diagnosis.

**Table 3:** Diagnostic accuracy results obtained from teledermoscopic evaluations of 1197 atypical pigmented facial lesions (aPFLs) in three different settings, stratified across levels of experience in dermoscopy and perceived level of difficulty.

Skill Level	Perceived Difficulty	N	Accuracy Setting 1 ^1^ (%)	Accuracy Setting 2 ^2^ (%)	Accuracy Setting 3 ^3^ (%)	Sensitivity (%)
<1 year	Easy	62	46.8 (34.0, 59.9)	64.5 (51.3, 76.3)	79.0 (66.8, 88.3)	46.2 (19.2, 74.9)
	Moderate	314	47.8 (42.1, 53.5)	62.1 (56.5, 67.5)	72.9 (67.6, 77.8)	54.1 (44.3, 63.7)
	Difficult	288	47.9 (42.0, 53.9)	55.6 (49.6, 61.4)	68.1 (62.3, 73.4)	59.6 (50.8, 67.9)
1–4 years	Easy	209	59.3 (52.3, 66.0)	74.2 (67.7, 80.0)	85.6 (80.1, 90.1)	65.2 (52.8, 76.3)
	Moderate	290	48.3 (42.4, 54.2)	57.6 (51.7, 63.3)	68.3 (62.6, 73.6)	49.6 (40.4, 58.8)
	Difficult	181	41.4 (34.2, 49.0)	49.7 (42.2, 57.2)	60.2 (52.7, 67.4)	50.6 (39.1, 62.1)
5–8 years	Easy	154	44.8 (36.8, 53.0)	59.1 (50.9, 66.9)	78.6 (71.2, 84.8)	26.2 (13.9, 42.0)
	Moderate	193	49.2 (42.0, 56.5)	58.0 (50.7, 65.1)	69.4 (62.4, 75.8)	40.8 (29.3, 53.1)
	Difficult	111	49.5 (39.9, 59.2)	55.0 (45.2, 64.4)	63.1 (53.4, 72.0)	58.3 (43.2, 72.4)
>8 years	Easy	219	59.8 (53.0, 66.4)	73.5 (67.1, 79.2)	82.6 (77.0, 87.4)	64.8 (52.5, 75.7)
	Moderate	238	49.2 (42.6, 55.7)	61.3 (54.8, 67.6)	71.0 (64.8, 76.7)	54.3 (44.3, 64.0)
	Difficult	186	36.6 (29.6, 43.9)	47.3 (40.0, 54.7)	57.5 (50.0, 64.7)	54.0 (43.0, 64.8)

^1^ LM + LMM vs. AN vs. PAK vs. SL vs. SK vs. SLK diagnosis. ^2^ LM + LMM diagnosis vs. PAK diagnosis vs. BKL + AN diagnosis. ^3^ LM + LMM diagnosis vs. Benign aPFLs diagnosis.

**Table 4:** Management per skill level, confidence, and rating.

		Close Dermoscopic Follow-Up	Reflectance Confocal Microscopy	Skin Biopsy
Experience	<1 year	276 (41.6%)	153 (23.0%)	235 (35.4%)
	1–4 years	298 (42.4%)	152 (22.4%)	230 (32.8%)
	5–8 years	209 (45.6%)	126 (27.5%)	123 (26.9%)
	>8 years	221 (34.4%)	166 (25.9%)	256 (39.8%)
Confidence	Not confident	120 (24.1%)	120 (36.1%)	198 (39.8%)
	Uncertain	170 (24.4%)	232 (33.2%)	296 (42.4%)
	Confident	714 (57.2%)	180 (14.8%)	350 (28.0%)
Rating	Easy	446 (69.3%)	49 (7.6%)	149 (23.1%)
	Moderate	424 (41.0%)	256 (24.7%)	355 (34.3%)
	Difficult	134 (17.5%)	292 (38.1%)	340 (44.4%)

**Table 5:** Updated management strategy by skill, confidence, and rating with histology.

Factor	Level	Histology	Close Dermoscopic Follow-Up	Reflectance Confocal Microscopy	Skin Biopsy
Skill Level	<1 year	Benign	216 (53.2%)	94 (23.2%)	96 (23.6%)
		LM + LMM	60 (23.3%)	59 (22.9%)	139 (53.9%)
	1–4 years	Benign	233 (53.8%)	94 (21.7%)	82 (18.9%)
		LM + LMM	65 (22.8%)	58 (20.3%)	148 (54.6%)
	5–8 years	Benign	158 (45.8%)	78 (22.6%)	109 (31.6%)
		LM + LMM	51 (31.7%)	48 (29.8%)	62 (38.5%)
	>8 years	Benign	174 (52.3%)	102 (30.6%)	104 (31.2%)
		LM + LMM	47 (19.7%)	64 (26.8%)	152 (63.6%)
Confidence	Not confident	Benign	90 (36.0%)	106 (42.4%)	54 (21.6%)
		LM + LMM	30 (14.3%)	74 (35.2%)	106 (50.5%)
	Uncertain	Benign	115 (30.2%)	136 (35.7%)	130 (34.1%)
		LM + LMM	55 (17.4%)	96 (30.3%)	166 (52.4%)
	Confident	Benign	576 (57.6%)	124 (12.4%)	121 (12.1%)
		LM + LMM	138 (32.4%)	59 (13.8%)	229 (53.8%)
Rating	Difficult	Benign	94 (22.6%)	176 (42.3%)	146 (35.1%)
		LM + LMM	40 (11.4%)	116 (33.1%)	194 (55.4%)
	Moderate	Benign	321 (51.2%)	153 (24.4%)	153 (24.4%)
		LM + LMM	103 (25.2%)	103 (25.2%)	202 (49.6%)
	Easy	Benign	366 (80.0%)	39 (8.5%)	54 (11.8%)
		LM + LMM	80 (41.0%)	24 (12.3%)	105 (53.8%)

## Data Availability

The raw data supporting the conclusions of this article will be made available by the authors on request.

## Appendix A

**Table A1:** Accuracy of the three settings (1: LM + LMM, AN, PAK, SL, SK, SLK—2: LM + LMM, PAK, BKL + AN—3: LM + LMM, Benign) and overall sensitivity for the assessment stratified across evaluators’ level of experience, confidence of the assessment, and perceived complexity of the lesion (rating).

Skill Level	Confidence	Rating	N	Accuracy Setting 1 ^1^	Accuracy Setting 2 ^2^	Accuracy Setting 3 ^3^	Sensitivity
<1 year	Not confident	Easy	2	50.0%	50.0%	50.0%	N.A.
		Moderate	53	45.3%	54.7%	69.8%	57.9%
		Difficult	186	43.5%	52.2%	67.7%	55.4%
	Uncertain	Easy	6	66.7%	83.3%	100.0%	N.A.
		Moderate	144	49.3%	62.5%	72.9%	56.4%
		Difficult	87	55.2%	60.9%	65.5%	62.5%
	Confident	Easy	54	44.4%	63.0%	77.8%	46.2%
		Moderate	117	47.0%	65.0%	74.4%	48.6%
		Difficult	15	60.0%	66.7%	86.7%	83.3%
1–4 years	Not confident	Moderate	20	30.0%	50.0%	55.0%	33.3%
		Difficult	87	33.3%	39.1%	50.6%	39.5%
	Uncertain	Easy	9	44.4%	55.6%	77.8%	33.3%
		Moderate	111	48.6%	53.2%	64.9%	44.0%
		Difficult	79	48.1%	59.5%	67.1%	61.8%
	Confident	Easy	200	60.0	75.0%	86.0%	66.7%
		Moderate	159	50.3%	61.6%	72.3%	56.3%
		Difficult	15	53.3%	60.0%	80.0%	57.1%
5–8 years	Not confident	Easy	1	100.0%	100.0%	100.0%	N.A.
		Moderate	15	33.3%	40.0%	53.3%	44.4%
		Difficult	30	50.0%	53.3%	60.0%	55.6%
	Uncertain	Moderate	71	43.7%	50.7%	64.8%	41.9%
		Difficult	67	49.3%	56.7%	65.7%	65.4%
	Confident	Easy	153	44.4%	58.8%	78.4%	26.2%
		Moderate	107	55.1%	65.4%	74.8%	38.7%
		Difficult	14	50.0%	50.0%	57.1%	25.0%
>8 years	Not confident	Easy	9	55.6%	66.7%	66.7%	50.0%
		Moderate	23	56.5%	60.9%	65.2%	45.5%
		Difficult	72	27.8%	40.3%	54.2%	50.0%
	Uncertain	Easy	2	50.0%	100.0%	100.0%	100.0%
		Moderate	52	38.5%	48.1%	57.7%	33.3%
		Difficult	70	38.6%	47.1%	54.3%	51.4%
	Confident	Easy	208	60.1%	73.6%	83.2%	65.2%
		Moderate	163	51.5%	65.6%	76.1%	62.9%
		Difficult	44	47.7%	59.1%	68.2%	63.6%

^1^ LM + LMM vs. AN vs. PAK vs. SL vs. SK vs. SLK diagnosis. ^2^ LM + LMM diagnosis vs. PAK diagnosis vs. BKL + AN diagnosis. ^3^ LM + LMM diagnosis vs. Benign aPFLs diagnosis.
